# Progressive Pernicious Anæmia

**Published:** 1882-08

**Authors:** Wm. Henry

**Affiliations:** Harmon Lee Co., Ill.


### ARTICLE IV.

### PROGRESSIVE PERNICIOUS ANÆMIA.

HARMON LEE CO., ILL., AUG. 3, 1882.

EDITORS CHICAGO MEDICAL JOURNAL AND EXAMINER:— The wife of Dr. Wm. Henry, after parturition, had no hæmorrhage more than an ordinary case of labor. The lochia the same as usual; perhaps a little more than the average, but not enoug[illegible]

to destroy the blood-making power. The extremities became œdematous; the color left the body; three weeks before death she looked as bloodless as a corpse. The ears were bloodless, and a gradual weakening of the powers of life came on. Sometimes a peculiar mold would come on the surface, which could be rubbed off, leaving under it on the skin a greenish-yellow color. For two weeks previous to death she had constant nausea, and sometimes vomiting. There was complete anorexia; no relish for food, but she had a great relish for ice and ice-water. No remedies that were used seemed to do any good, as in ordinary anæmia. I used tonics, diuretics, preparation of iron; but all to no purpose. She seemed to die of slow asthenia. The disease progressed in spite of all treatment. From the description Dr. Henry Hartshorne gives of that disease in Reynolds' System of Medicine, this seems to be a clear case of progressive pernicious anæmia. During the whole time there was no perceptible rise in temperature, nor much increase in the circulation; nothing but a gradual changing of the blood to water. The urine was about normal in quantity; sometimes light in color; at others dark. The bowels were constipated at times, and at others regular. The spleen and liver seemed somewhat enlarged; tongue pale, as in ordinary anæmia.

At times she complained of a sound in her head as if it were water, with strange noises. No emaciation of the body, but it seemed as plump as in health. Her babe was eight weeks old when she died; it is still living and doing well.

WM. HENRY, M.D.

---

THE following is the arrangement of the staff of the Woman's Hospital of the State of Illinois since the recent reorganization:

Dr. W. H. Byford, Dr. J. H. Etheridge, Dr. Daniel T. Nelson, Dr. H. P. Merriman, Dr. E. O. F. Roler, Dr. E. Warren Sawyer, Dr. A. M. Davenport.

Consulting Physicians. Dr. J. Ramsey Flood, Dr. D. R. Brower.

